# Pharmacy-based non-communicable diseases screening has high potential for reach: lessons from Rwanda

**DOI:** 10.3389/fpubh.2025.1684688

**Published:** 2025-10-21

**Authors:** Calvin Chiu, Melsa Omaya, Francois Uwinkindi, Dudu Tang, Samuel Mwubahamana, Francine Miller, Josh Ruxin, Matthew Rehrig, Jean-Baptise Mazarati, Ida Alexandra de Cordier, Jenny X. Liu, David Zapol

**Affiliations:** ^1^Institute for Health & Aging, University of California San Francisco, San Francisco, CA, United States; ^2^Goodlife Access, Kigali, Rwanda; ^3^Rwanda Ministry of Health, Rwanda Biomedical Center, Kigali, Rwanda; ^4^School of Public Health, University of California Berkeley, Berkeley, CA, United States

**Keywords:** healthcare delivery, global health, screening, non-communicable diseases, pharmacy, Rwanda

## Abstract

Low and middle-income countries are entering an epidemiological transition where non-communicable diseases (NCDs) increasingly contribute to disease burden. While Rwanda has one of the most robust public health systems in sub-Saharan Africa, rapid urbanization is leading to an emerging middle-class population who are at high risk for NCDs, but who also underutilize preventive care. The Goodlife Access pilot program in Rwanda screened over 9,000 individuals for diabetes, hypertension, and obesity over 18 months for free; 83% of individuals showed abnormal screening results, 90% of whom had no prior diagnosis. Pharmacy-based screenings effectively reached a high-risk, underserved population and highlights the potential of community pharmacies to amplify NCD diagnoses. While one-third of those screened returned for confirmatory testing at the pharmacy, more work is needed to bolster re-engagement, including addressing fear of confirmatory testing and potential medical treatment costs. Given the successful pilot, Goodlife Access is expanding access to NCD screening while incorporating community-based health insurance and user donations to enhance sustainability.

## 1 Introduction

Non-communicable diseases (NCDs) such as diabetes, cardiovascular conditions, and cancers, accounted for 74% of all deaths worldwide in 2023 (1). Alarmingly, 77% of these NCD-related deaths and 86% of premature deaths occurred in low- and middle-income countries (LMICs) where mortality is disproportionately high given limited and fragmented care (2). Early detection and timely treatment can effectively improve prognosis and reduce resource burden for individuals and healthcare systems alike (3). However, overstretched public healthcare systems in LMICs often do not offer NCD information or screenings (4, 5), and thus are poorly positioned to meet the changing burden of disease.

Community pharmacies have emerged as a viable response to this challenge. Conveniently located and often the first point of contact for care-seeking, pharmacies offer a unique opportunity to expand access to NCD screening, not just among consumers with the ability to pay out-of-pocket, but also for low-income and other vulnerable populations that public healthcare systems have limited capacity to reach (6, 7). However, there is limited evidence regarding the feasibility and acceptability of pharmacy-based NCD screening programs. Pilot studies in Ghana, South Africa, Kenya and Tanzania have been small (<300 patients) and follow-up with patients after initial screening was limited (8–10). A recent review in Ethiopia found that community pharmacy professionals provided screening and management of noncommunicable diseases, but faced barriers such as limited training and resources, lack of reimbursement, and inadequate integration into the healthcare system (11). We present lessons learned from a large pilot program reaching over 9,000 people with free routine screening for diabetes, hypertension, and obesity at a chain of 13 private pharmacies in Rwanda–among the first programs of its kind in sub-Saharan Africa.

## 2 Pharmacy-based NCD screening pilot

### 2.1 Screening

In a public-private partnership endorsed by the Rwanda Ministry of Health, the Goodlife Access program offered free screenings for diabetes, hypertension, and obesity to over 9,000 individuals at for-profit community-based private pharmacies between January and December 2024. Trained nurses conducted free screenings for the three focal health conditions at three pharmacy screening sites. Initially, the program offered screening to customers at the pharmacy or neighboring shops and clinics before adding active community outreach and demand creation at monthly community service events and sporting events.

After registering and giving consent, participants were screened for diabetes (blood glucose test using a Droga glucometer), hypertension (blood pressure reading), and obesity (body mass index calculated from height and weight measurements), and informed about risk factors and prevention strategies. Those with normal screening results were informed about the need for repeated screenings every 3 months. Those with elevated risk indicators received tailored NCD education about modifiable exposures and behaviors for reducing risk of developing NCDs, and scheduled a follow-up confirmatory retest at the pharmacy.

### 2.2 Follow-up

Participants with a confirmatory retest appointment were instructed to present for resting after fasting for at least 8 h. At the time of the appointment, a trained nurse measured blood pressure and administered a blood glucose test. Participants with repeated elevated results were referred to a clinic for a comprehensive consultation and potential diagnosis. Referrals were tailored to the participant’s individual situation based on their location and socioeconomic status, the latter defined by receiving insurance through Mutuelle de Santé, Rwanda’s national community-based health insurance for low-income people not otherwise covered by government or private employers. A subset of participants who screened positive for diabetes or hypertension and insured through Mutuelle de Santé were enrolled in an intensive follow-up intervention which assessed participants’ ability to pay for clinical care and medication and provided health coaching to marginalized and at-risk individuals.

## 3 Methods

First, using administrative data from the program, we computed descriptive statistics on participant demographics (age, sex, insurance coverage) and screening results from the program. We defined an abnormal result for diabetes (glucose <70 mg/dL or >120 mg/dL), hypertension (systolic ≥140 mmHg or diastolic ≥90 mmHg) and obesity (BMI > 24.9 or <18.4) in accordance with standard thresholds adopted by the World Health Organization. We also computed descriptive statistics on previous risk factors and diagnoses, whether participants were repeat visits, and where participants who required follow-up confirmatory testing intended to seek care. We plotted bar charts comparing test positivity rates by age, sex and insurance coverage. We tested whether these differences were statistically significant using a likelihood ratio test on the significance of age, sex and insurance coverage, respectively, from a multivariate logistic regression models on test positivity, reporting 95% confidence intervals and *p*-values accordingly. Second, using follow-up survey data from 300 randomly sampled participants, we computed descriptive statistics on whether participants completed a follow-up visit after being referred, and if not, reasons for not doing so. Third, we synthesized lessons learned from the implementation experience based on discussions and reflections from the implementation team. Note that since the data capture participants seeking care at a pharmacy, we focus our interpretation of results among this subpopulation without further generalization.

## 4 Results

Table 1 shows participant demographics and screening results from the program. We found that 70% of participants had at least one high or elevated screening results: 24% for elevated glucose, 24% for elevated blood pressure, and 58% for high body mass index, respectively (12). Further, 18% had at least one risk factor associated with these conditions, with lack of exercise (11%), unhealthy diet (11%) and alcohol use (7%) being the most common. The program reached individuals across a wide age range and was particularly effective in reaching low-income and other vulnerable populations–65% were covered by Mutuelle de Santé, and 17% were uninsured. Critically, 90% had no prior diagnosis of diabetes, hypertension, or obesity, showing that the program effectively reached high-risk populations with no prior awareness of their risk for these conditions. Of those who required follow-up confirmatory testing, 53% expressed interest in visiting a private clinic neighboring the pharmacy chain and 11% a public health facility, but 33% had no planned follow-up.

**Table 1:** Descriptive statistics.

*N* = 9,635 (%)	*N* (%)
Age	
Below 30	2,222 (23.10%)
30–44	4,062 (42.20%)
45–59	2,560 (26.60%)
60 and above	738 (7.70%)
Missing	53 (0.60%)
Sex	
Male	5,960 (63.60%)
Female	3,418 (35.50%)
Missing	257 (2.70%)
Insurance coverage (covered by)	
Mutuelle	6,252 (64.90%)
RSSB	1,512 (15.70%)
Private	258 (2.70%)
None	1,613 (16.70%)
Prior diagnosis	
Diabetes	400 (4.20%)
Hypertension	554 (5.70%)
Other (non-diabetes hyperglycemia/hypotension)	4 (0.00%)
None	8,677 (90.10%)
Repeat visit (Yes)	1955 (20.30%)
Screened positive	
Diabetes (glucose <70 mg/dL or >120 mg/dL)	2,208 (23.80%)
Hypertension (systolic ≥140 mmHg or diastolic ≥90 mmHg)	2,325 (24.40%)
Obesity (BMI > 24.9 or <18.4)	5,200 (58.40%)
Any	6,747 (70.10%)
Follow-up	
Goodlife clinic	1,312 (53.00%)
Other private clinic	52 (2.10%)
Public clinic	277 (11.20%)
Traditional healer	11 (0.40%)
No planned follow-up	825 (33.30%)
Risk factor	
Alcohol use	682 (7.10%)
Family history	356 (3.70%)
Lack of exercise	1,061 (11.00%)
Overweight obese	623 (6.50%)
Hormonal contraceptives	39 (0.40%)
Smoking	44 (0.50%)
Unhealthy diet	1,082 (11.20%)
At least one NCD risk factor	1,686 (17.50%)

**Figure 1:**
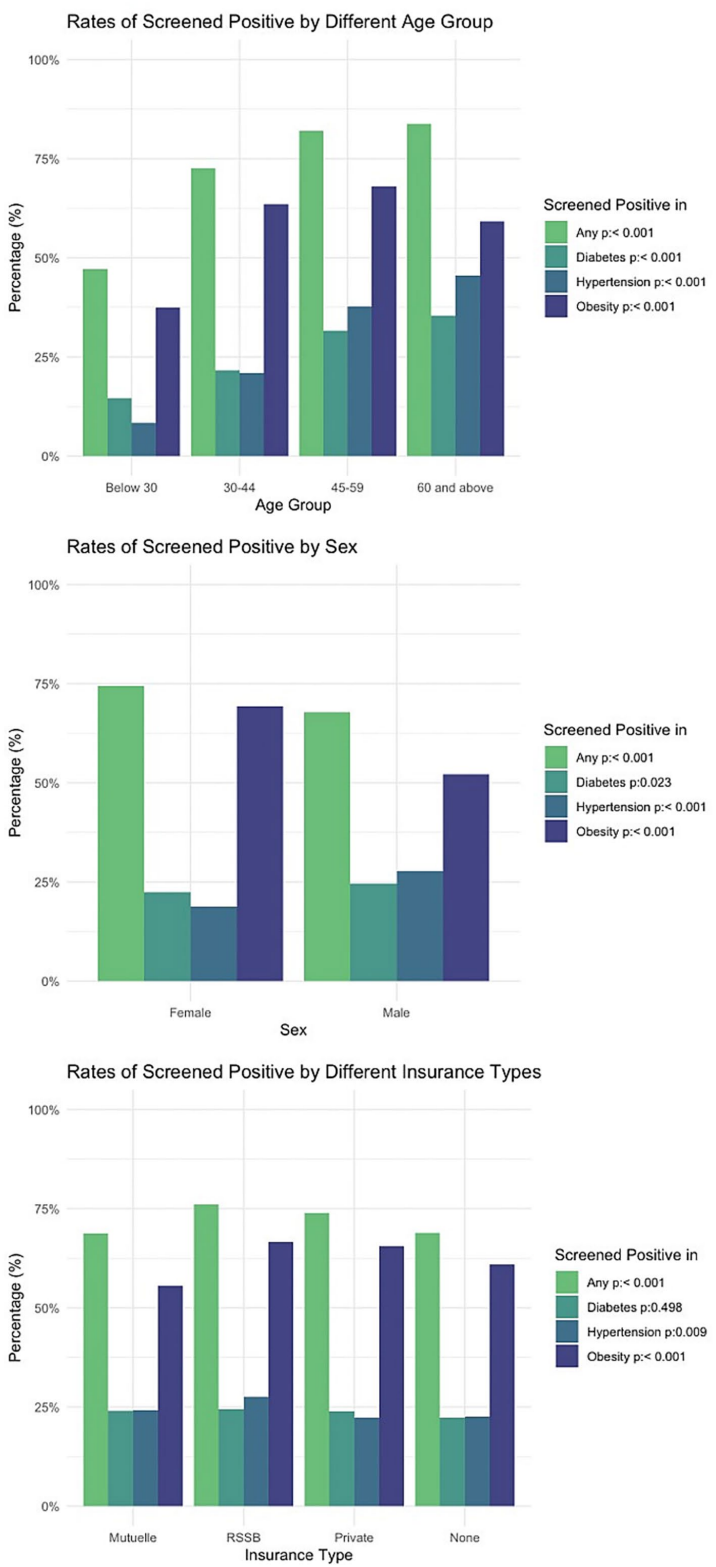
Percentage of screened positive, stratified by age, sex, insurance type.

Figure 1 shows test positivity rates by age, sex, and insurance coverage. As expected from differences in underlying disease burden, test positivity rates were higher among older participants for all three conditions. Compared to female participants, males had lower screen-positive rates for obesity (52% vs. 69%, OR = 0.40, 95% CI, 0.36–0.44, *p* < 0.001), but higher rates for hypertension (28 vs. 19%, OR = 1.47, 95% CI, 1.32–1.64, *p* < 0.001); rates for diabetes were similar (25% vs. 22%, OR = 1.04, 95% CI, 0.93–1.15, *p* = 0.50). Screen-positive rates differed by insurance type: compared to those with other insurance coverage (none, Mutuelle, or private) those insured by the Rwandan national social security fund (RSSB) had higher rates for obesity (67% vs. 58%, OR = 1.49, 95% CI, 1.31–1.70, *p* < 0.001) but similar rates for diabetes (24% vs. 24%, OR = 0.96, 95% CI, 0.84–1.11, *p* = 0.603) and hypertension (28% vs. 24%, OR = 1.07, 95% CI, 0.94–1.23, *p* = 0.318).

In follow-up surveys with 300 randomly sampled participants, one-third completed a follow-up visit after being referred. Over 15% of participants did not follow-up on abnormal screening results due to fear, anxiety, or a perceived lack of importance. Individuals expressed “anxiety or fear that the disease will recur” and “anxiety about taking medication [in the] long-term.”

### 4.1 Lessons learned

The Goodlife Access pilot showed that pharmacy-based screening for diabetes, hypertension and obesity in Rwanda was a highly promising strategy for expanding the reach of early NCD detection. Our initial experience has garnered particular insight for expanding reach and sustainability of this emerging public-private partnership.

1. Patients preferred pharmacy-based NCD screening due to the convenient locations and accessibility vis-a-vis extended hours and much shorter wait times. Among the 300 participants randomly surveyed, most found out about the program when passing through the screening site and pharmacy (79%), compared to word of mouth (17%) and other advertising (flyers and social media). Thus, the pharmacy setting allows for an opportune moment to screen for NCDs when customers already have health on their minds (13).
2. Pharmacy-based NCD screening reached high-risk, low-income individuals – populations who would not have otherwise screened at a public health facility– and at greater convenience. Notably, 15% of visits occurred after hours (after 5:30 p.m. when public facilities typically close), and utilization after-hours was somewhat higher (85%) compared to during regular business hours (81%) among low-income participants.
3. Although confirmatory testing was also offered at pharmacy sites, more is needed to bolster re-engagement and retesting. The program attempted to address fear of receiving a confirmed diagnosis or committing to sustained treatment by providing health education on NCDs and health-promoting behaviors but more work is needed to optimize and simplify referral pathways for users, including overcoming stigma and fear associated with clinical healthcare services (14).
4. Future efforts to implement the program at scale will require additional financing to ensure sustainability. To demonstrate proof-of-concept, Goodlife Access pilot program offered free screenings, the cost of which was partially offset by participant donations and private donors during the pilot. Sustainable financing, such as reimbursement via the Rwandan national social security fund (RSSB), could be considered.
5. Stakeholder engagement was critical to developing and implementing the pilot program. Given limited capacity in the public healthcare sector and need to leverage private sector resources, the Ministry of Health engaged multisectoral partners (public, private, and non-profit organizations including Rwanda Biomedical Center, RSSB, and Goodlife Access) to develop the program in alignment policy priorities (the Ministry of Health’s Strategic Priorities Plan for 2023–2025).

## 5 Conclusion

Leveraging a network of pharmacies, the Goodlife Access program demonstrated the feasibility of pharmacy-based screenings for NCDs in LMICs. Pilot results showed that pharmacy-based NCD screenings were both effective and potentially scalable. Building on this success, the program intends to expand its scope to include breast, cervical, and colon cancer screening.

With increasing urbanization and reliance on private-sector services over under-resourced public systems, integrating NCD screening into pharmacies presents an effective means of addressing gaps in access. However, private-sector involvement is often limited by low reimbursement rates, while public healthcare systems remain underfunded, necessitating innovative cross-sector partnerships to bridge these gaps. This includes potentially charging co-pays from patients, the creation and integration with national insurance, or other public-private partnerships (6, 7, 15). While pharmacy-based diabetes and cholesterol screening has been found to be cost-effective in other settings (16, 17), future research to examine the cost-effectiveness of more comprehensive pharmacy-based NCD screening is required. Policy reforms that expand scopes of practice and encourage pharmacists to use their clinical skills as part of the primary healthcare team are also needed (18).

## Data Availability

The datasets presented in this article are not readily available because the datasets for this study cannot be shared publicly without restrictions due to legal restrictions under the Rwandan Data Privacy Act (Law No. 058/2021 of 13/10/2021) governing the storage, transfer, and disclosure of data without regulatory authorization. Qualified researchers may request access to the data by contacting the corresponding author. Data sharing will require submission of a data access request to the Rwandan National Cyber Security Authority and a signed data use agreement in accordance with Rwandan regulations. Requests to access the datasets should be directed to Calvin Chiu, Calvin.Chiu@ucsf.edu.
